# Structural Biology and Electron Microscopy of the Autophagy Molecular Machinery

**DOI:** 10.3390/cells8121627

**Published:** 2019-12-12

**Authors:** Louis Tung Faat Lai, Hao Ye, Wenxin Zhang, Liwen Jiang, Wilson Chun Yu Lau

**Affiliations:** 1 School of Life Sciences, Centre for Cell and Developmental Biology and State Key Laboratory of Agrobiotechnology, The Chinese University of Hong Kong, Shatin, New Territories, Hong Kong, China; 2 CUHK Shenzhen Research Institute, The Chinese University of Hong Kong, Shenzhen 518057, China

**Keywords:** plant autophagy, autophagosome, cryo-electron microscopy, single-particle analysis, autophagy-related

## Abstract

Autophagy is a highly regulated bulk degradation process that plays a key role in the maintenance of cellular homeostasis. During autophagy, a double membrane-bound compartment termed the autophagosome is formed through de novo nucleation and assembly of membrane sources to engulf unwanted cytoplasmic components and targets them to the lysosome or vacuole for degradation. Central to this process are the autophagy-related (ATG) proteins, which play a critical role in plant fitness, immunity, and environmental stress response. Over the past few years, cryo-electron microscopy (cryo-EM) and single-particle analysis has matured into a powerful and versatile technique for the structural determination of protein complexes at high resolution and has contributed greatly to our current understanding of the molecular mechanisms underlying autophagosome biogenesis. Here we describe the plant-specific ATG proteins and summarize recent structural and mechanistic studies on the protein machinery involved in autophagy initiation with an emphasis on those by single-particle analysis.

## 1. Introduction

Macroautophagy (henceforth known as autophagy) is an evolutionarily conserved eukaryotic “self-eating” process for the degradation of damaged proteins and organelles, protein aggregates, and invading pathogens [1,2,3]. Due to its vital role in nutrient recycling and the maintenance of cellular homeostasis, deregulation of autophagy has been tightly linked to the pathogenesis of a variety of human diseases including cancer, neurodegenerative disorders, and metabolic diseases. In plants, autophagy is essential for growth and development, immunity, as well as cellular responses to biotic and abiotic stresses [4,5,6]. Unlike animals, plants as sessile organisms rely on autophagy to confer tolerance and survival from various unfavorable environmental conditions including climate change and pollution. Impairment in plant autophagy is associated with early leaf senescence, hypersensitivity to nitrogen starvation, and reduced crop yield. During autophagy, a double membrane-bounded vesicle, termed the autophagosome, is formed to engulf unwanted cytoplasmic materials and subsequently fuses with the lysosome/vacuole leading to the degradation of the engulfed cargos [7]. Upon autophagy initiation, an isolated membrane known as the phagophore is formed at diverse membrane sites such as endoplasmic reticulum (ER) subdomains, mitochondria-ER contact sites, the ER-Golgi intermediate compartments, the plasma membrane, and Golgi apparatus [8,9,10,11,12]. Then, the phagophore expands into a cup-shaped structure through the acquisition of lipids and eventually seals to complete the formation of the autophagosome. Being a highly dynamic process, autophagosome biogenesis can be divided into four stages including initiation, nucleation, expansion, and maturation [13,14], all of which are highly dependent on and tightly regulated by a dedicated set of protein machineries known as the autophagy-related (Atg/ATG) proteins (denoted by the letters Atg and ATG in yeast and mammals/plants, respectively). Most of the core ATG genes are conserved from yeast to plants to humans, and together they consist of six distinct functional groups: the ATG1 kinase complex, the class III phosphoinositide 3-kinase (PI3K) complex, the transmembrane protein ATG9, the ATG2-ATG18 complex, as well as proteins belonging to the ATG8- and ATG12-conjugation systems [15,16,17]. Compared to their yeast and mammalian counterparts, plants contain some additional homologs or plant-specific components, the function of which remain to be explored [18,19,20,21,22,23]. It is noteworthy that biochemical, structural, and functional studies of plant ATG proteins are severely hampered by the difficulty in protein production, a challenge often encountered when working in plant proteins in general [24]. There exists several technologies dedicated to producing proteins in plants, including the *Agrobacterium*-mediated transient gene expression [24], chloroplast transformation [25], and stable transformation that integrates foreign genes into the plant nuclear genome [26]. Unfortunately, both the transient expression and the chloroplast transformation are of only limited use at present owing to the inability to either express proteins of large size or in sufficient quantity, which is particularly important for structural studies. The long time frame required for generating stable transgenic plants also renders this approach impractical for in vitro applications. As a result, large-scale production of plant proteins today still largely relies on heterologous expression in bacteria, yeast, and mammalian cells.

The goal of structural biology is to determine the three-dimensional (3D) arrangement of molecules in order to understand the protein chemistry at the atomic level. This provides indispensable information for dissecting the detailed molecular mechanism of a biological process. Until now, X-ray crystallography has been tremendously successful in the structural biology field, where more than 85% of structures deposited in the Protein Data Bank were determined by X-ray crystallography. Provided that the protein of interest can be crystallized, structures at atomic resolution can be routinely obtained by this technique, regardless of the size and complexity of the protein. However, generating protein crystals is notoriously difficult, especially for integral membrane proteins and multi-subunit protein complexes, contributing to a bottleneck for structure determination of these types of proteins [27,28,29]. Still, structural investigation of autophagy machinery has progressed for more than a decade, and most of the structures were predominantly determined by X-ray crystallography (Table 1) [30,31,32,33,34,35,36,37,38,39,40,41,42,43,44,45,46,47,48,49,50,51,52,53,54,55,56,57,58,59,60,61,62,63,64,65,66,67,68,69,70,71,72,73,74]. In recent years, single-particle analysis and cryo-electron microscopy (cryo-EM) have attracted considerable attention in the field, and they have matured into robust methods for solving structures at high resolution without the need for crystallization. Moreover, proteins with different compositional and conformational heterogeneity can be studied by cryo-EM [75], which is suitable for studying protein complexes with dynamic and intrinsically disordered properties such as ATG proteins.

Despite extensive research over the past decades, our knowledge on the underlying molecular mechanisms of autophagosome biogenesis remains far from complete [76,77]. Recent studies using single-particle electron microscopy (EM) has contributed remarkable progress in the structural elucidation of several ATG proteins [46,67,73,78,79]. Here we focus on the structural biology aspect of the autophagosome biogenesis with emphasis on studies by single-particle EM and discussed the structure-function relationship of the core ATG proteins involved in autophagy initiation.

## 2. The ULK1/ATG1 Complex

**Figure 1:**
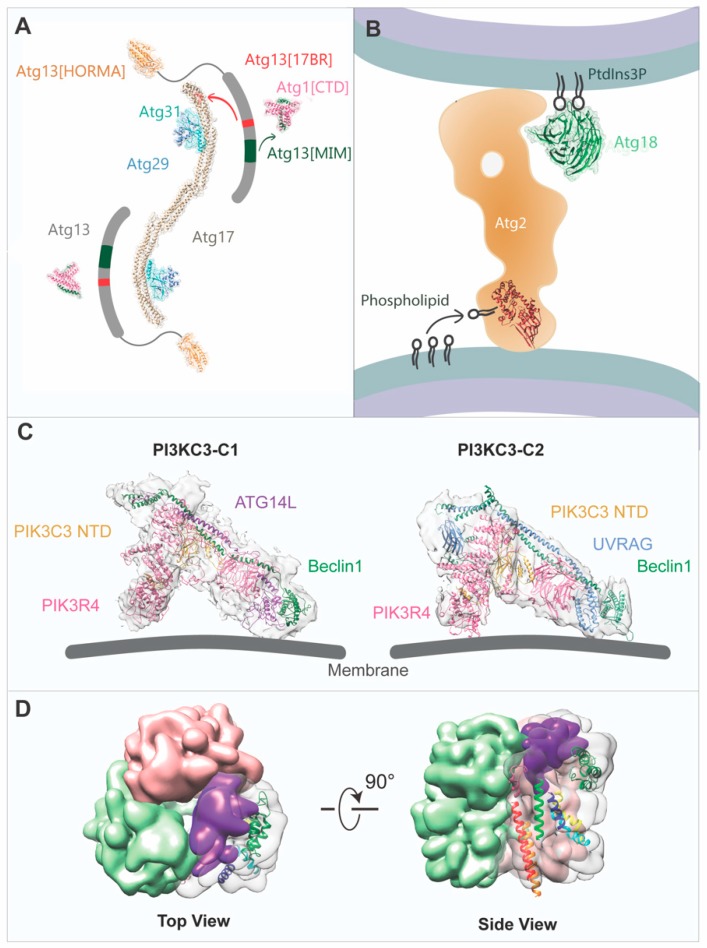
Models of ATG core machinery built from electron microscopy (EM) and crystallographic data. (**A**) Mapping of yeast Atg1 kinase complex formation. Atg13[CTD] (grey) links the Atg1[CTD] (PDB:4P1N, pink) to the Atg17-Atg31-Atg29 subcomplex (PDB: 4P1W, Atg17 in tan, Atg31 in cyan, Atg29 in blue). The Atg13 HORMA domain (orange, PDB: 4J2G) locates to the outward of the N terminus of Atg17. The MIM domain of Atg13 (shown in dark green) and the Atg17-binding region of Atg13 (shown in red) interact with Atg1[CTD] and Atg17, respectively. (**B**) Model of Atg2-18 complex for autophagosome formation. Crystal structures of the N-terminal region of Atg2 (PDB:6A9E, in red) and Atg18 (PDB:5LTD, in green) are fitted into the model. (**C**) Cryo-EM structures of PI3KC3-C1 (EMD-6785) and C2 (EMD-6787) docked with the built atomic model and yeast C2 model (PDB: 5DFZ, VPS34 CTD excluded), respectively. The atomic model of C1 generated from homology modelling of yeast VPS34, VPS15, VPS30, and ATG14 structures (PDB: 5DFZ) was fitted into the density map. (**D**) Surface view of *Arabidopsis* ATG9 trimer map (EMD-9681) with transmembrane helixes cytoplasmic regions docked into single protomer. The C-terminal region is colored in purple.

Activation of the ULK1/ATG1 complex is considered to be first step of autophagosome biogenesis and is directly regulated by the nutrition sensing machineries TOR complex [80] and AMPK complex [81,82]. Induced by starvation, autophagy signals are initially transmitted to the ULK1/ATG1 kinase complex, which is responsible for the recruitment of downstream regulators. While the assembly of the budding yeast Atg1 complex appears to be regulated by the TOR signaling pathway, the mammalian ULK1 complex is a stable complex regardless of nutritional status [83,84]. The human ULK1 complex consists of the ULK1 protein kinase, the FAK family kinase interacting protein of 200 kDa (FIP200), and the Hop/Rev7/Mad2 (HORMA) domain-containing proteins ATG13 and ATG101 (Figure 1). While ULK1 and ATG13 have orthologs in the yeast Atg1 complex, four ATG1 and two ATG13 paralogs have been found in *Arabidopsis* (Table 2) [17,20,32,47,56,74,77,85,86,87,88,89,90,91,92,93,94]. Furthermore, multiple canonical ATG1 loci have been identified by scanning the available plant genome sequences, including three in maize (*Zea mays*), four in poplar (*Populus trichocarpa*), and two each in rice (*Oryza sativa*), suggesting that ATG1 proteins are widely distributed throughout the plant kingdom and may have functional redundancy. Plants typically have more than one ATG13, phenotypical analysis to *Arabidopsis atg13a/b* mutant plants suggests ATG13a and ATG13b have redundant functions in autophagy. Like ATG1, orthologs of ATG13 have been found in a number of angiosperms. The yeast scaffolding protein Atg17 is predicted to function as FIP200 in mammals [95], but the budding yeast *Saccharomyces cerevisiae* possesses no Atg101 and its Atg1 complex subunits Atg29 and Atg31 have no orthologs in mammals [96,97]. In *Arabidopsis*, ATG17 and ATG101 have been identified, but how they function as regulatory and/or scaffolding subunits is largely unknown and awaits future investigation [98].

According to previous studies using X-ray crystallography, the yeast Atg17-Atg31-Atg29 trimer forms a crescent-shaped stable complex with stochiometric ratio of 2:2:2 [30]. The Atg17 monomer is composed of four α-helices folded in a crescent coiled-coil with a length of 194 Å and curvature of ≈100 Å in radius. Atg31 is comprised of N-terminal β-sheets sandwiching a β-strand of Atg29, and a C-terminal helix binding to Atg17, bridging Atg17 and Atg29 in the trimer. Atg17-Atg31-Atg29 further forms a dimeric complex in vitro [30,85]. Several possible forms of dimerization were observed in the crystal lattice, but only one form is in excellent agreement with the structural coordinates calculated from experimental solution small-angle X-ray scattering data. This dimeric form was later on supported by an independent negative stain EM study, revealing that the dimeric Atg17-Atg31-Atg29 complex exhibits an S-shaped arrangement [32]. According to the crystal structure, dimerization is mediated via hydrophobic residues (Leu355, Ile358, Leu 359, Leu366, and Ile369) at the C-terminus of Atg17. Interestingly, two-dimensional (2D) EM analysis has revealed that the Atg17 alone displayed a variety of conformations, instead of exhibiting a stable S-shaped structure. Although Atg29 and Atg31 are not directly involved in Atg17 dimerization, the study has also revealed a potential regulatory role of Atg29 and Atg31 as their interactions with Atg17 constrain the flexibility of the Atg17 dimer and stabilize the S-shaped conformation. Unlike Atg17 that can fold into stable helical bundles, Atg1 and Atg13 possess an intrinsically disordered region (IDR), which hinders structural studies on the complete Atg1 pentameric complex using X-ray crystallography [33]. Nevertheless, the interactions of Atg13 with Atg1 and Atg17 have been revealed by two crystal structures published in 2014: the tandem microtubule interacting and transport (MIT) domains within the C-terminal region of Atg1 in complex with the minimal Atg1-binding domain MIM of Atg13 and Atg17-Atg29-Atg31 in complex with the minimal Atg17-binding region (17BR) of Atg13 [33]. Consistent with these crystal structures, subsequent single-particle EM and crosslinking coupled with mass spectrometry studies also supported the binding of the C-terminal regions of Atg1 and Atg13 to the distal ends of the crescent Atg17 in the Atg1 pentameric complex [35], and that Atg17 likely interacts with C-terminal Atg29 IDR and C-terminal Atg31 IDR, both of which are missing from the Atg17-Atg29-Atg31 crystal structure.

On the contrary to the yeast Atg1 complex, structural information of the intact ULK1 complex in mammals has remained unexplored until a recent study reported the EM analysis of the complex [46]. The FIP200 N-terminal domain (NTD) was found to be a dimer. It exhibits in a highly flexible C-shaped structure, with the N-termini at the tips and the C-termini at the center of the structure, observed under negative stain EM. The FIP200 NTD is also able to bind to ATG13-ATG101 and ULK1 via a FIP200 segment (443–450 aa) to ATG13 middle region (363–460 aa) (ATG 13 MR) and FIP200 (319–326 aa) to ULK1-EAT, respectively. The interactions of FIP200 NTD with ATG13-ATG101 and ULK1 further stabilize its C-shaped structure and reduce structural flexibility of the NTD. Furthermore, the ULK1 complex is a asymmetric complex with a FIP200 NTD:ATG13:ATG101:ULK1 ratio of 2:1:1:1, where ATG13-ATG101 and ULK1 were only found on one tip of the FIP200 NTD dimer. High-resolution structural analysis by cryo-EM has resolved the FIP200 NTD-ATG13 MR complex to 12–15 Å resolution. The resolution of the map was likely hindered by the conformational heterogeneity of FIP200 NTD, suggested by the fact that cryo-EM maps with different degrees of curvature were observed after 3D classification. Together with the crystallographic study of the FIP200 Claw domain, it is proposed that FIP200 both serves as a hub for ULK1 complex formation during autophagy initiation and binds to p62 to promote the recruitment of cargo to the isolation membrane during aggrephagy [44].

Other structures of the partial components of the ULK1/ATG1 complex has also been studied in the past decade, including the kinase ATG1/ULK1/2 and the ATG13-ATG101 complex. Among them, the ATG13-ATG101 is one of the complexes that has been extensively studied [34,41,43]. The ATG13-ATG101 complex in both fission yeast and mammal displays a conserved architecture with ATG13 and ATG101 in C-Mad2 and O-Mad2 conformations, respectively. The WF finger of the ATG101, which is required for recruiting downstream regulators including WIPI1 and ZFYVE1, folds from an open to a closed conformation upon the binding to ATG13 [41,42]. In addition, the interaction between ATG13 and ATG101 induces the conversion of a β-strand to α-helical structure within the ATG101 C-terminal region, which further mediates the recruitment of PI3KC3 complex by direct interaction [43].

## 3. The PI3KC3 Complex

Immediately downstream of the ATG1 complex is the tetrameric autophagy-specific class III phosphatidylinositol 3-kinase (PI3KC3) complex (Figure 1). Its main function is to produce phosphatidylinositol 3-phosphate (PI3P), which is the key phospholipid required for the recruitment of downstream autophagy machinery necessary to drive the expansion of the autophagosomal membrane [99,100,101]. In yeast and mammalian cells, there exist two different PI3KC3 complexes, PI3KC3-C1 and PI3KC3-C2 (Table 2). The PI3KC3-C1 complex is essential for autophagy nucleation, whereas the PI3KC3-C2 complex is involved in both autophagosomal membrane expansion and non-autophagic processes including Golgi-ER retrograde transport, endocytic trafficking, and endosome maturation. These two complexes share a common core consisting of the Vacuolar Protein Sorting 15 (VPS15, Vps15 in yeast), phosphatidylinositol 3-kinase catalytic subunit Vacuolar Protein Sorting 34 (VPS34, Vps34 in yeast), and Bcl2-interacting protein 1 (BECN1, Vps30/Atg6 in yeast), but are distinguished by a fourth component, ATG14L (Atg14 in yeast) for PI3KC3-C1 [102,103] and UV radiation resistance-associated gene protein (UVRAG, Vps38 in yeast) for PI3KC3-C2 [104,105]. Correspondingly, these subunits can be found in plants and are expected to function similarly as their orthologs in yeast and mammals. Notably, with the exception of *Arabidopsis*, ATG14 is commonly absent in other plants, suggesting that there may be alternative proteins to complement the function of ATG14 [5,106]. ULK1/ATG1 complex activates PI3KC3-C1 via phosphorylation of BECN1 at Ser15, increasing its activity in generating PI3P at the phagophore [87].

Although PI3KC3 has been extensively studied for many years, most structural studies have focused on individual domains, for example, the Vps15 WD repeat domain [107], VPS34 with inhibitors [58,65,66], Atg6/PECN1 BARA domain 155 [55], Beclin 1 CC domain [63], conformational flexibility within and between domains [108,109], as well as individual domains in complex with regulatory proteins such as the complex of the BECN1 BH3 domain and BCL2 homologs [59,60,61,62]. The first 3D structure of the intact human PI3KC3-C1 complex was solved by negative stain EM and provided a glimpse of the structure at low resolution, uncovering its V-shaped architecture [67]. Subsequently, the structure of the yeast PI3KC3-C2 complex was determined by X-ray crystallography to 4.4 Å resolution [56]. This structure exhibits an overall similar architecture to that of the C1 complex at low resolution. In this higher resolution structure, Vps15 contacts and restricts the activation loop of Vps34 to inhibit its activity. The Vps15 kinase domain is also likely in the inactive state since its long activation loop protrudes into the ATP binding site, thus preventing ATP binding. The two coiled-coils from both Vps30/Atg6 and Vps38 wind up into a heterodimer in a parallel manner. Together with the Vps15 WD domain, they form one arm of PI3KC3-C2. On the other side, the kinase domains and the helical domains from both Vps15, Vps34 and Vsp34 C2 domain interact in an antiparallel manner to form the other arm. The Vsp34 C2 domain is sandwiched in the center of the complex, and it acts as a hub engaging with all other subunits. This domain is also important for tight interaction between Vps15-Vps34. In addition, the authors proposed a model for the PI3KC3-C2 complex on the membranes with the tips contacting the lipids, with one arm via Vps34 and Vps15 and the other via the Vps30 β-α repeated, autophagy-specific (BARA) domain. In a later cryo-EM study, the structures of the human PI3KC3-C1 and -C2 complexes were determined at sub-nanometer resolution (≈9 Å) [70]. EM analysis of the human PI3KC3-C1 complex on lipid monolayers revealed that the complex also interacts with the membranes via the tips of the two arms similar to the yeast PI3KC3-C2 complex. One major difference is that the ATG14L CTD in the human PI3KC3-C1 complex contacts the membrane on one tip instead of Vps30 as proposed for the PI3KC3-C2 complex in yeast. Besides the intact complexes, structures of the human PI3KC3-C2 bound to the PI3KC3-C2 binding domain (PIKBD) of its endogenous inhibitor Rubicon [79] and the human PI3KC3-C1 bound to its positive regulator Nuclear Receptor Binding Factor 2 (NRBF2) [73] have also been recently determined by cryo-EM. Rubicon was found to bind to the BECN1 BARA domain, likely inhibiting the interaction of PI3KC3-C2 with the membrane. On the other hand, the binding site of NRBF2 was mapped to the base of the V-shaped complex, and NRBF2 binding promotes the transition to its active conformation whereby the highly dynamic VPS34 kinase domain is liberated from the Vps15 kinase domain and is positioned in a precise geometry to catalyze the phosphatidylinositol phosphorylation reaction on the membrane substrate.

## 4. The ATG2-ATG18/WIPI Complex

ATG2-ATG18/WD-repeat protein interacting with phosphoinositides (WIPI) axis is the downstream effector of PI3P produced by the PI3KC3 complex [110,111,112,113] (Figure 1). While there exists only one homolog for Atg2 and Atg18 in yeast, there are two ATG2 homologs in mammalian cells, ATG2A and ATG2B [114], as well as four ATG18 homologs, known as WIPI 1–4 [115,116] (Table 2). WIPI4 shows a stronger binding capacity with either ATG2A or ATG2B than the other three WIPIs [51]. The conserved aromatic H/YF motif within the C terminus of ATG2 is important for ATG2-WIPI complex formation. In addition to autophagosome formation, mammalian ATG2 is also crucial for regulating the morphology and dispersion of lipid droplets [114]. In plants, eight potential Atg18 proteins were revealed from the *Arabidopsis* genome by BLAST searches, named AtATG18a–AtATG18h [91]. So far, only ATG18a has been shown to be required for autophagosome formation in plants.

ATG2-WIPI/ATG18 localizes at the edge of phagophore and is required for the expansion of the phagophore [93,114,117,118]. The recruitment of ATG2-WIPI to phagophore, where PI3P is enriched, is mediated through the binding of two PI3P by the motifs in blades 5 and 6 of the seven-bladed β-propeller folded WIPI, as inferred from crystal structure of Hsv2 (a homolog of Atg18) [47,48,49,119,120]. Despite knowing that ATG2-WIPI functions at the ER-phagophore contact site, the exact role of ATG2-WIPI has remained a mystery for decades, until recent structural studies revealed that ATG2 possesses lipid-transfer properties [50,53]. ATG2 is the largest protein in ATG core machinery, consisting of ≈1600–2300 residues among eukaryotes and harboring the conserved Chorein_N, ATG_C, and ATG2_CAD domains [52]. Negative stain EM and domain labelling with MBP have uncovered that human ATG2A is a rod-like protein, with the N-terminal domain located at one end and the CAD domain located on the opposite end adjacent to WIPI4 [51,52]. As expected, the overall shape of yeast Atg2-Atg18 shares high similarity with the rod-shaped mammalian ATG2-WIPI complex, suggesting functional conservation of this complex across species. Besides, ATG2A alone can tether highly curved membrane at both ends without causing fusion of two membrane sources. It has been hypothesized that the CAD tip of ATG2 together with WIPI4 attached to the PI3P-enriched omegasome, while the N-terminal tip of ATG2 associates with another membrane source to facilitate lipid transfer for phagophore expansion. The flexible attachment of WIPI4 to ATG2A may facilitate its tethering to the omegasome. Strikingly, a recent crystallographic study has elucidated that the Atg2 N-terminal domain possesses a hydrophobic pocket, which can capture phosphatidylethanolamine (PE) molecules [50]. The structure strongly suggests that Atg2 mediates direct lipid transfer between two membrane sources, which is also confirmed by in vitro lipid transfer assay. In addition, a long internal cavity extended from the N-terminal domain to the opposite end of ATG2 is observed in the cryo-EM structure, giving rise to the possibility that lipid molecule is transferred from one end to another through this cavity of ATG2 [53]. Yet, high-resolution structural information about the remaining part of ATG2 is needed to fully elucidate the detailed mechanism of lipid transfer.

## 5. The Transmembrane Protein ATG9

ATG9 is the only integral transmembrane protein in the core ATG machinery (Figure 1; Table 2). ATG9 localizes to the TGN, late endosomes, and tubular-vesicular membrane clusters with a 30–60 nm diameter, termed the ATG9 vesicles/compartments [121,122,123,124]. Upon autophagy initiation, ATG9-vesicles/compartments are recruited and tethered to the phagophore assembly sites in yeast or the omegasomes in plants and mammals. ATG9/Atg9 is phosphorylated by ULK1/Atg1 and dynamically interacts with the autophagosomal membrane without being stably integrated into the autophagosome. The source of the membrane required for autophagosome biogenesis remains a long-standing question in the field of autophagy. ATG9 vesicles/compartments have been suggested to provide an essential membrane source for phagophore nucleation and expansion [124,125,126]. Nevertheless, the exact function of ATG9 is still largely elusive due to the lack of structural information about this protein. ATG9 is predicted to possess six transmembrane helices, and predominantly disordered N- and C- terminal regions facing the cytoplasm. In yeast, the Atg9-core domain lacking the N- and C-terminal regions has been shown to interact with Atg17 via the crescent-forming helix α4, which also harbors the Atg31-binding site (Table 2) [127]. Due to its transmembrane and intrinsically disordered nature, structural studies on ATG9 are notoriously challenging. Nevertheless, our group has successfully determined the structure of the trimeric Arabidopsis ATG9 by cryo-EM at 7.8 Å resolution, revealing the overall architecture and domain organization [73]. Although no atomic model is available for ATG9, we have generated the first putative model of the protein by integrating co-evolutionary information and homology modelling approaches. Our model provides structural information about the orientation of the six transmembrane helices, the N-terminal region and a large loop (middle loop) within the transmembrane core. Of note, the yeast Atg1 complex binds to the middle loop of Atg9 via Atg17 and possibly tethers the Atg9-vesicles in cells [127]. Thorough analysis of the SAXS data and revisiting the crystal lattice packing observed between two *Lachancea thermotolerans* Atg17-Atg31-Atg29 dimers suggests that the Atg1 pentamer is capable of forming tetramers in solution, potentially allowing for scaffolding of a cluster of Atg9-containing vesicles at the PAS for phagophore nucleation [36]. In addition, our structure reveals that ATG9 self-interacts with adjacent protomers via transmembrane and cytoplasmic regions, in contrast with the previous finding that the self-interaction of Atg9 is mediated solely via the cytoplasmic C-terminal regions [128]. Based on the 2D class averages of ATG9 cryo-EM images, flexible cytoplasmic regions are observed in both monomers and dimers, but are absent from trimers, suggesting that disordered cytoplasmic regions fold into an ordered structure upon trimerization. We speculate that the cytoplasmic regions of ATG9 protomers from opposing vesicles/compartments interact during autophagy, pulling them toward each other, and eventually result in membrane fusion, through a process which likely requires the help of additional regulators. In addition, ATG9 is known to be regulated by ULK1/Atg1 via phosphorylation at the cytoplasmic regions, which is important for both trafficking [129] and binding to ATG2-ATG18 [130]. Studying the conformational change of ATG9 upon phosphorylation will be crucial for unveiling its functional role in autophagy. Our model of ATG9 provides a framework for further biochemical and cell biological studies of ATG9 mutants to dissect the mechanistic relationship of ATG9 and ULK1/Atg1 and the ATG2-ATG18 complexes during phagophore initiation and nucleation.

## 6. Concluding Perspectives

High-resolution structural information is imperative for the detailed understanding of ATG proteins in regulating the initiation of autophagy. Extensive past studies in yeast and mammalian cells have been shaping the model of autophagosome formation at unprecedented details. However, in plants, knowledge of the mechanism of autophagy is still in its infancy. Furthermore, plants have a much higher diversity of ATG gene families and carry plant-specific homologs, further complicating the investigation of ATG complexes and derivation of a well-defined model for plant autophagosome biogenesis. Resolving the structure of the *Arabidopsis* ATG9 represents a milestone in the field of plant autophagy. Nevertheless, many questions remained to be addressed such as the functional significance of the ATG9 trimer, the mode of interaction with other ATG proteins, and the underlying regulatory mechanisms. Determining cryo-EM structures of ATG9 at higher resolution will certainly help to address these questions. Single-particle cryo-EM has revolutionized the field of structural biology and has become one of the most powerful and robust techniques for determining structures and studying the conformational dynamics of a wide variety of protein complexes. In addition, electron tomography has been recently applied to the study of intracellular processes in plants with spectacular success, enabling the visualization of unperturbed cellular ultrastructures including autophagosome-related tubules inside cells [20,131]. Undoubtedly, EM will continue to contribute rapidly to the field of autophagy and promises to bring groundbreaking advances in autophagy research in the forthcoming future.

## Figures and Tables

**Table 1:** List of structures of the core autophagy-related (ATG) machinery involved in autophagy initiation determined by various structural biology techniques. *Lt*: *Lachancea thermotolerans; Km: Kluyveromyces marxianus; Sp: Schizosaccharomyces pombe; Kl: Kluyveromyces lactis. Saccharmyces cerevisae* are denoted as Yeast.

Complex	Component	Origin	Method	Resolution (Å)	Year	References
ATG1 complex	Atg17-Atg31-Atg29	Yeast	X-ray	3.05	2012	[30]
	Atg13 HORMA	Yeast (*Lt*)	X-ray	2.3	2013	[31]
	Atg17-Atg31-Atg29	Yeast	Negative stain	37	2013	[32]
	C-terminal region of Atg1(MIT)-ATG13MIM	Yeast (*Km*)	X-ray	2.2	2014	[33]
	Atg17-Atg29-Atg31-Atg13(17BR)	Yeast (*Lt*)	X-ray	3.2	2014	[33]
	Atg101-Atg13	Yeast (*Sp*)	X-ray	3	2015	[34]
	Atg1 complex (Atg17-Atg31-Atg29 and Atg17-Atg31-Atg29-Atg1[CTD]-Atg13[CTD])	Yeast	Negative stain	/	2015	[35]
	Atg1-Atg13 and Atg17-Atg31-Atg29 subcomplexes and the Atg1 complex	Yeast (*Kl*)	SAXS	/	2015	[36]
	Atg17–Atg29–Atg31-Atg13(17BR)-Atg13(17LR)	Yeast (*Lt*)	X-ray	3.2	2016	[37]
	Atg17	Yeast (*Sp*)	Negative stain	/	2017	[38]
	Kinase domain of ULK1 with inhibitor	Mammal	X-ray	1.88	2015	[39]
	Kinase domain of ULK1 with inhibitor	Mammal	X-ray	1.74	2015	[40]
	ATG13 HORMA-ATG101 HORMA	Mammal	X-ray	2.2	2015	[41]
	ATG101	Mammal	X-ray	1.9	2015	[42]
	ATG101-ATG13HORMA	Mammal	X-ray	2.5	2018	[43]
	FIP200 CTR	Mammal	X-ray	3.2	2019	[44]
	Kinase domain of ULK2 with inhibitor	Mammal	X-ray	2.5	2019	[45]
	ULK1 complex	Mammal	Cryo-EM	12-15	2019	[46]
ATG2-18	Hsv2 (ATG18 homolog)	Yeast (*Km*)	X-ray	2.6	2012	[47]
	Hsv2 (ATG18 homolog)	Yeast	X-ray	3	2012	[48]
	Hsv2 (ATG18 homolog)	Yeast (*Kl*)	X-ray	3	2012	[49]
	N-terminal domain of Atg2	Yeast (*Sp*)	X-ray	3.2	2019	[50]
	ATG2B(human)-WDR45(rat)	Mammal	Negative stain	/	2017	[51]
	ATG2A-WIPI4	Mammal	Negative stain	/	2018	[52]
	ATG2A	Mammal	Cryo-EM	15	2019	[53]
PI3K complex	VPS15 WD repeat domain	Yeast	X-ray	1.8	2009	[54]
	VPS30 BARA domain	Yeast	X-ray	2.3	2012	[55]
	PI3KC3-C2	Yeast	X-ray	4.4	2015	[56]
	ATG38 C-terminal domain	Yeast	X-ray	2.2	2016	[57]
	VPS15-VPS34	Yeast	Negative stain	28	2016	[57]
	VPS34 with inhibitors	Drosophila	X-ray	2.9–3.5	2010	[58]
	Bcl-X_L_-Beclin 1 BH3	Mammal	X-ray	2.5	2007	[59]
	Bcl-X_L_-Beclin 1 BH3	Mammal	NMR	/	2007	[60]
	M11-Beclin1 BH3	Mammal	X-ray	2.3	2008	[61]
	M11-Beclin 1 BH3	Mammal	X-ray, NMR		2008	[62]
	Beclin 1 CC domain	Mammal	X-ray	1.9	2012	[63]
	Beclin 1 ECD domain	Mammal	X-ray	1.55	2012	[64]
	VPS34 with PIK-III	Mammal	X-ray	2.8	2014	[65]
	VPS34 with SAR405	Mammal	X-ray	2.9	2014	[66]
	PI3KC3-C1	Mammal	Negative stain	27.5	2014	[67]
	Beclin 1 FHD domain	Mammal	X-ray	1.95	2016	[68]
	Beclin 1 CC domain	Mammal	X-ray	1.46	2016	[69]
	ATG14 CC domain with/without Beclin 1 CC domain	Mammal	SAXS	/	2016	[69]
	PI3KC3-C1 with NRBF2	Mammal	Negative stain	/	2016	[57]
	PI3KC3-C1 with NRBF2	Mammal	Negative stain	/	2017	[70]
	PI3KC3-C1 and PI3KC3-C2	Mammal	Cryo-EM	8.5 (C1) and 8.6 (C2)	2017	[71]
	Beclin 1-UVRAG CC domain	Mammal	X-ray	1.9	2018	[72]
	PI3KC3-C1 with NRBF2 dimer	Mammal	Cryo-EM	6.6	2019	[73]
ATG9	ATG9	Plant	Cryo-EM	7.8	2019	[74]

**Table 2:** The core ATG machinery involved in autophagy initiation in yeast, mammals, and plants.

	Yeast (*Saccharomyces cerevisiae*)	Mammal (*Homo sapiens*)	Plant (*Arabidopsis thaliana*)	Function in Autophagy	Protein Interactions*	Reference
ATG1 complex	Atg1	ULK1	ATG1a ATG1b ATG1c ATG1t	S/T kinase	*S. cerevisiae* 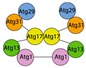	[17,32,77,85,86]
	Atg13	ATG13	ATG13a ATG13b	Regulatory subunit	*H. sapiens* 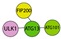	
	Atg11 Atg17 Atg29 Atg31	FIP200 ATG101	ATG11 ATG101	Scaffold and regulatory	*A. thaliana* 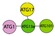	
Class III PI3K complex I	Vps34	VPS34	VPS34	PI kinase	*S. cerevisiae* 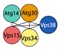	[56,86,87,88]
	Vps15	VPS15	VPS15	Scaffold		
	Vps30/Atg6	BECN1	ATG6	Regulatory subunit	*H. Sapiens* 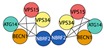	
	Atg14	ATG14	ATG14a ATG14b	PAS targeting (N.C. in plant)		
	Atg38	NBRF2	-	Activator		
ATG9 vesicle	Atg9	ATG9A/B	ATG9	Phagophore formation and expansion (Autophagosome progression and closure in plant)	*S. cerevisiae* 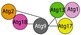	[20,73,90,91,92]
					*H. Sapiens* 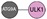	
					*A. thaliana* 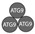	
ATG2-ATG18 complex	Atg2	ATG2A/B	ATG2	PAS targeting and lipid binding	*S. cerevisiae* 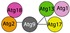	[47,92,93,94,95]
	Atg18	WIPI1/2	ATG18a-h			

Abbreviations: ATG, autophagy-related gene; VPS, vacuolar protein sorting-associated protein; PI, phosphatidylinositol; WIPI, WD repeat domain phosphoinositide-interacting protein; PAS, pre-autophagic structure; N.C., not characterized. *Protein-protein interaction is based on protein structural study and biochemical data.
